# Restoring Fertility in Sterile Childhood Cancer Survivors by Autotransplanting Spermatogonial Stem Cells: Are We There Yet?

**DOI:** 10.1155/2013/903142

**Published:** 2013-01-03

**Authors:** Robert B. Struijk, Callista L. Mulder, Fulco van der Veen, Ans M. M. van Pelt, Sjoerd Repping

**Affiliations:** Centre for Reproductive Medicine, Women's and Children's Hospital, Academic Medical Centre, University of Amsterdam, 1105 AZ Amsterdam, The Netherlands

## Abstract

Current cancer treatment regimens do not only target tumor cells, but can also have devastating effects on the spermatogonial stem cell pool, resulting in a lack of functional gametes and hence sterility. In adult men, fertility can be preserved prior to cancer treatment by cryopreservation of ejaculated or surgically retrieved spermatozoa, but this is not an option for prepubertal boys since spermatogenesis does not commence until puberty. Cryopreservation of a testicular biopsy taken before initiation of cancer treatment, followed by *in vitro* propagation of spermatogonial stem cells and subsequent autotransplantation of these stem cells after cancer treatment, has been suggested as a way to preserve and restore fertility in childhood cancer survivors. This strategy, known as spermatogonial stem cell transplantation, has been successful in mice and other model systems, but has not yet been applied in humans. Although recent progress has brought clinical application of spermatogonial stem cell autotransplantation in closer range, there are still a number of important issues to address. In this paper, we describe the state of the art of spermatogonial stem cell transplantation and outline the hurdles that need to be overcome before clinical implementation.

## 1. Introduction

Childhood cancer, defined as cancer occurring before the age of 14, is an increasingly prevalent disease that affects many children across the globe. More than 12.000 children in the USA alone are diagnosed with cancer each year [1]. In Europe, the incidence of childhood cancer is estimated to be 139 per million children [2]. Highly effective cancer treatments have led to a spectacular increase in life expectancy in these children, from a 60% 5-year survival rate in the late 1970s to an 80% 5-year survival rate in 2002 [3]. It is estimated that currently 1 in 250 young adults is a survivor of childhood cancer [4]. 

Given this success in pediatric oncology, long-term adverse side effects of cancer treatment have become of increasing importance [5]. One of the most prevalent long-term side effects of cancer treatment in boys is infertility. Cancer treatment regimens such as alkylating agents and radiation therapy [6, 7] destroy the small pool of spermatogonial stem cells (SSCs) in the prepubertal testis. SSCs are the progenitors of male gametes and thus critical for sperm production and the ability to father offspring. Already present at birth, SSCs reside on the basal membrane of the seminiferous tubules in the testes. Before puberty SSCs do not develop into sperm, but after onset of puberty they will maintain spermatogenesis throughout the rest of a man's life. 

Loss of spermatogonial function impairs the generation of functional gametes thereby leading to infertility [8]. Rates of gonadal dysfunction in childhood cancer survivors are variable and depend on dose and type of treatment [9], ranging from a mean 17% azoospermia in patients after treatment of different types of tumors [10] to 82% after treatment for Hodgkin disease [11]. Prepubertal patients are regularly too young to fully understand the profound impact of therapy on their reproductive capacity, but two-thirds of parents whose prepubertal boy has been diagnosed with cancer would agree to freeze a testicular biopsy if a future therapy could lead to potential restoration of spermatogenesis [12, 13]. An interview among long-term childhood cancer survivors between 19–37 years old revealed that most of the participants wish to have genetically own children in the future [14] and becoming infertile due to cancer treatment is a reduction in quality of life for these patients [15]. Not only does cancer treatment impose devastating effects on one's ability to have children, childhood cancer survivors also suffer from psychological effects due to their disease history and some even experience problems in attracting a partner because of being infertile [14]. 

Until cancer treatment can exclusively target tumor cells, infertility among these boys will remain an important long-term consequence. Oligozoospermic adult cancer patients may consider intracytoplasmic sperm injection (ICSI) of ejaculated sperm into an oocyte and azoospermic patients may theoretically benefit from testicular sperm extraction (TESE) [16] followed by ICSI if spermatozoa are found [17]. Those survivors who are completely sterile (i.e., when no spermatozoa are found upon TESE) have no way of achieving a pregnancy from their own genetic material. Men that develop cancer before adolescence do not have functional spermatozoa as spermatogenesis does not commence until puberty and they cannot be helped by TESE/ICSI either. Needless to say, there is substantial need for a technique that safeguards or restores fertility in these long-term cancer survivors.

SSC autotransplantation may be a way to restore the spermatogonial stem cell pool after cancer treatment, thereby leading to life-long spermatogenesis and the chance to achieve pregnancy. Transplantation of SSCs was first described in mice in 1994, generating full spermatogenesis in an otherwise infertile recipient mouse and functional sperm leading to donor-derived offspring [18]. This achievement boosted research on SSC functionality and has led to major advancements in unraveling SSC biology that will hopefully pave the way to future clinical implementation (see Table 1).

Based on the mouse transplantation model, the theoretical way to restore reproductive potential in human male childhood cancer survivors is to cryopreserve a testicular biopsy before cancer treatment and to transplant cells from the biopsy back into the testis when that patient is cured from cancer and expresses the wish to have children [19, 20]. Briefly, SSC transplantation can be achieved by ultra-sound guided needle injection of testis cell suspensions into the rete testis of a recipient as was shown to work in several large animal models and in human testis *ex vivo *[21–23] (see Section 5). Besides this proposed SSC transplantation therapy model (SSCT), other experimental technological approaches to tackle infertility include testis tissue grafting [24, 25], *in vitro *production of spermatozoa from SSCs [26], and derivation of male germ cells from induced pluripotent cells (iPS) [27], but these approaches are still in the very early experimental phase. 

**Figure 1:**
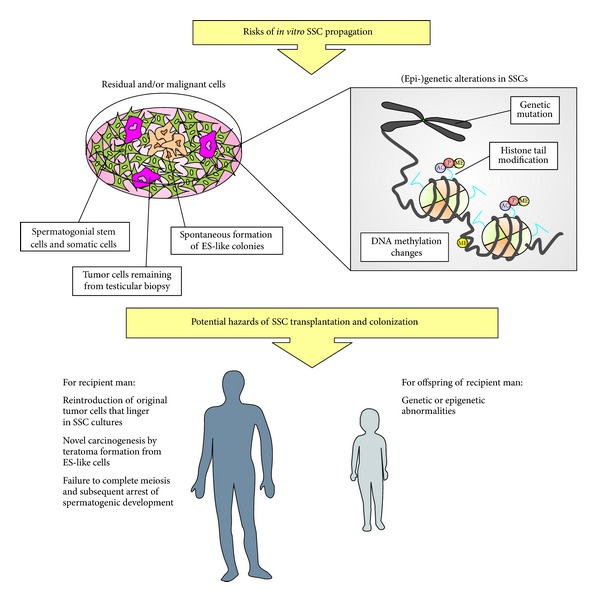
Potential risks of *in vitro* SSC propagation and subsequent SSC transplantation. In *in vitro* propagated SSCs cultures derived from a patient testis biopsy, there is the risk that unwanted cells, such as lingering tumor cells from the patient or spontaneously formed colonies of ES-like cells, are present in the material used for transplantation. Structural integrity of propagated SSCs might be affected due to culture conditions, either on the genetic or the epigenetic level. Alterations that arise *in vitro* can potentially influence the health of the recipient patient or the offspring of that patient.

The most critical steps in bringing SSCT to the clinic involve *in vitro* propagation to increase the limited number SSCs from a small testis biopsy, assessment of genetic and epigenetic stability during SSC propagation *in vitro*, elimination of possible remaining malignant cells and investigation of the health of offspring generated after autotransplantation (summarized in Figure 1). In this review, we focus on the current state of the art of SSCT and we provide a stepwise description of what has been achieved concerning these matters. We will also outline the obstacles that need to be overcome before SSCT can be implemented in the clinic as a means to restore fertility in sterile childhood cancer survivors.

## 2. Proliferation of SSCs *In Vitro*

As is the case for stem cells in many tissues, the fraction of SSCs compared to surrounding somatic cells is relatively low. In mice, SSCs represent only around 0,03% of all testicular cells [28]. To obtain enough SSCs for transplantation, the few SSCs originating from prepubertal testis biopsies need to be expanded artificially to repopulate an adult testis. Clinicians would need to compensate for the larger testicular volume in which cells are transplanted back, especially considering that an adult human testis is approximately 60 fold larger than a prepubertal testis biopsy. Successful long-term *in vitro* proliferation of SSCs was first demonstrated in mouse [29] and more recently in adult men and prepubertal boys [30, 31]. When cultured for 64 days, the number of SSCs increased over 18,000 fold in a human testicular cell culture system [31]. After culture, human spermatogonia were still detectable as shown by the expression of markers for undifferentiated germ cells *PLZF, ITG*α*6, *and *ITG*β*1* [30]. Upon transplantation in immunodeficient mice, these cells were able to migrate to the niche in the seminiferous tubule, as was shown by the presence of the human marker COT-1. Xenotransplantation of human SSCs to the mouse testis using cells of an early and late time point in culture shows that artificial propagation of SSCs is possible in men. 

Expansion of SSCs in an *in vitro* culture system would ideally resemble the *in vivo* situation as closely as possible. In the *in vivo* situation, a complex niche environment exists where SSCs and somatic supporting cells interact to establish essential intracellular signaling. A number of factors have been identified that are required for stem cell maintenance (e.g., EGF, LIF, GDNF, and bFGF) [32]. Artificial mimicking of the niche environment is very difficult, because there are numerous factors that orchestrate the interaction between SSCs and somatic cells and most of them are only poorly characterized. Usage of a “feeder layer” (a layer of somatic cells, often inactivated mouse embryonic fibroblasts) is considered essential for successful propagation of SSCs [29, 33]. Growth of spermatogonia on a feeder layer will result in three-dimensional aggregates termed “clusters,” that contain multiple cell types including SSCs [34]. In the mouse germ line stem cell culture systems, animal-derived serum, and a feeder layer are used to mimic the *in vivo *environment [29].

For future human clinical application, a clinical grade medium would preferably not contain any serum derived from animals due to possible zoonotic or xenotoxic effects. The use of somatic cells present in the testis biopsy might maintain SSCs and circumvent the use of exogenous feeder cells. On the other hand, one can imagine that culturing in media lacking (animal-derived) serum [41] or certain growth factors [42] might impact on SSC function, possibly leading to reduced germ line potential [43]. Interfering with culture conditions is a double-edged sword with on one hand the improvement of propagation efficiency by addition of certain factors and on the other hand the possibility of altering SSC functionality because of those same additions. 

## 3. Genetic Stability of SSCs *In Vitro*

Since SSCs are the only stem cells in the adult male body capable of eventually transmitting information to a subsequent generation, it is crucial that these cells are genetically identical to their *in vivo* counterparts. Alterations to the genome are well known to change cellular phenotypes and can lead to a spectrum of genetic diseases [44–46]. These alterations, for example, translocations, small deletions or duplications, base pair mutations or copy number variations (CNVs) can be the direct result of an instable genome.

Artificial propagation of mammalian cells in an *in vitro* environment has been shown to cause instability of the genome [47, 48]. For instance, *in vitro* culture of murine hematopoietic stem cells, which normally reside in hypoxic bone marrow, induced chromosomal instability associated with relatively high oxygen tension in culture [49]. Such a chemical stressor could cause DNA damage induced by reactive oxygen species and account for loss of genomic integrity. Another example of an external stressor influencing genome stability is temperature. For proper functioning, testicular tissue normally requires a slightly lower temperature of 2–4°C below body temperature [50]. Surprisingly, SSCs are typically propagated at 37°C [29–31], while it is known that elevated temperature of the testis is associated with decreased testis weight, decreased testis viability, and induction of DNA damage in spermatozoa [51]. How exactly the genome becomes instable is not known, but the mode of action is perhaps similar to the way carcinogenic events cause large chromosomal changes *in vivo* [52]. Rather than being driven by an active process, structural mutations may also arise spontaneously, as was shown for cultured hematopoietic stem cells [53]. Mutations that escape normal DNA repair are clonally expanded in an *in vitro* environment and will persist in every newly formed cell. 

Cultures of mouse SSC show a normal euploid karyotype after 139 passages (~2 years of culture), indicating that they remain genetically highly stable even after prolonged exposure to *in vitro* culture conditions [54]. This suggests that SSCs possess a unique mechanism to prevent or repair genetic changes. However, in the same study, loss of telomeres has been observed. Although it would take many cell divisions before telomeres reach a critically short length so that cell senescence would be induced, senescent cells no longer divide, which might result in too few stem cells for transplantation in the case of SSCT. Conversely, it is known that telomere length is highly variable within a pool of male germ line stem cells and that germ cells are very tolerable to either high or low telomere lengths [55]. Whether genetic alteration or telomere shortening occurs in cultures of human SSCs has not been studied yet. 

## 4. Epigenetics in Cultured SSCs 

Besides changes in the genetic code itself, alterations in the epigenetic state of a cell can also occur as a result of environmental stressors [56, 57]. Epigenetics refers to the study of epigenetic traits, defined as *“stably inherited phenotypes resulting from changes in a chromosome without alterations in the DNA sequence”* [58]. One of the main functions of epigenetic modification is to establish differential gene expression by regulating transcription factor binding capacity to promoter regions, either via DNA methylation or chromatin modification. As it is well established over recent years, DNA methylation is closely intertwined with surrounding chromatin subunits and chromatin-related proteins [59] and distortion of the epigenetic landscape is associated with major diseases such as cancer [60, 61]. Some examples of *in vitro* aberrations of DNA methylation in stem cells come from studying cultured human mesenchymal stromal cells, which were shown to have significant changes in methylation when comparing a late passage to an early passage [62]. In this case, activation/repression of homeobox genes by changes in DNA methylation caused mesenchymal stromal cells to undergo senescence. Furthermore, altered DNA methylation in MSCs is correlated with repressive histone marks, which also leads to senescence [63]. 

In light of SSC transplantation, propagation of cells and transplantation procedures could serve as trigger for genetic and epigenetic changes, which may affect the health of SSC derived offspring (see Section 7). In a study comparing sperm derived from SSCs in grafts versus sperm derived from SSCT, no DNA methylation changes were found between these groups, but transplantation-derived sperm showed some variation in histone 4 acetylation [64]. Aberrant histone acetylation at this stage in development might have limited significance because in humans 85–95% of all histones are replaced by protamines to ensure proper packaging of DNA before delivery [65]. The small percentage of histones that do persist reside on HOX-gene promoters, miRNA genes, and imprinted genes. It has not been investigated if a change in histone modifications hampers functionality of the sperm. Culturing mouse testicular cells in medium containing GDNF and/or LIF does not alter methylation of the paternal imprinted *H19* locus, indicating that growth factors do not alter DNA methylation *per se *[66]. Long-term (>2 years) culture of mouse germ line cells also does not alter DNA methylation as was shown by combined bisulfite restriction analysis (COBRA) of five selected imprinted genes [54]. It should be noted that analysis of DNA methylation is often limited to a selection of imprinted genes, which may lead to a biased underestimation of epigenetic changes on the genome level. There is a need for experiments that will include all CpG sites in the genome and that will shed light on the true epigenetic status of a cell instead of a selected proportion of the genome. DNA methylation or histone modification has not been investigated in human cultures of SSCs.

## 5. Colonization of Cultured SSCs after Transplantation

It is essential that propagated SSCs maintain their ability to migrate to the niche and colonize the seminiferous tubules of a recipient testis upon transplantation. Nearly two decades ago murine testis-derived cells were transplanted in the testis of recipient mice and achieved colonization in 70% of the mice [18]. If a sufficient number of cells were transplanted, progeny could be generated harboring the same haplotype as the donor male mice. Since then, many groups have reported colonization of mouse SSCs and homing to a niche in the testes of mice [29, 67–71]. Others managed to perform successful homologous transplantations in pig [72], bull [73], non-human primate [21, 23] and recently zebrafish [74, 75]. Xenotransplantation to mouse recipients has been performed using dog [76], hamster [77], and bull [78–80] SSCs. 

Building on the data gathered in animal models, several labs reported successful human SSC xenotransplantation using either uncultured cell suspensions [38] or *in vitro *propagated SSCs [30, 31]. Human SSCs can be cultured for long periods of time while maintaining their ability to migrate to their niche upon transplantation. However, xenotransplanted human SSCs cannot undergo spermatogenesis but will rather divide a limited number of times and steadily decrease in number. An explanation why human SSCs cannot undergo full spermatogenesis in a mouse host environment is that postnatal primate SSCs, including human SSCs, are different from other species in terms of the expression of several spermatogonial markers like *POU5F1* (also known as *OCT-3/4*) [81] and *MAGE-A4* [82]. However, the spermatogenic arrest seen upon xenotransplantation could also be a result of phylogenic differences between the donor and recipient species [38, 83, 84] as is seen for many other non-primate species [76, 79]. Even so, xenotransplantation of SSCs is considered the only reliable bioassay at present to test for SSC functionality. In continuation of successful SSC xenotransplantation, homologous transplantation of primary SSCs has been demonstrated to initiate spermatogenesis in non-human primates [23]. In a recent publication, functional sperm was derived from both adult and prepubertal infertile rhesus macaques after autologous SSCs transplantation [21]. Not only was regeneration of spermatogenesis shown, but sperm derived from transplanted animals was also capable of fertilizing rhesus oocytes producing embryos ranging from four-cell stage to blastocyst with confirmed donor parental origin in 8,6% of embryos. There is a single report of SSC transplantation in humans in which a testicular cell suspension from cryopreserved testicular tissue was transplanted in 7 men [85]. Apart from this single study, other clinical attempts to reintroduce spermatogenesis in humans have not been described.

## 6. Remaining Tumor Cells in Testis Biopsy

Concerns have been raised about the potential presence of malignant cells in a biopsy taken from a patient that was diagnosed with cancer. Patients diagnosed with nonsolid tumors would be at high risk for this, because there is a chance that infiltrated tumor cells in the testis biopsy may linger in the *in vitro* culture and end up in the cell population used for transplantation. The most commonly diagnosed nonsolid tumor in prepubertal individuals is acute lymphoblastic leukemia (ALL), which infiltrates the testis in approximately 30% of cases [86]. For solid tumors that do not originate in the testis, the risk of nonintentional transplantation seems limited because solid tumors rarely metastasize to the testis [87]. 

Attempts to remove malignant cells from testicular cells have been limited. By sorting uncultured murine testicular cells mixed with leukemic cells for MHC-I^+^/CD45^−^, leukemic cells could be successfully separated from germ cells [88] and after transplantation of these cells to a recipient mouse, no leukemia was observed. Others have tried to reproduce these findings but did not succeed in completely removing malignant cells from the transplanted cell population [89]. Inoculation of T-lymphoblast cells with prepubertal primate testis cells still has a remainder of 0.1% of tumor cells after FACS sorting for CD90^+^(THY-1^+^) and CD45^−^, and the remaining tumor cells were able to form tumors after transplantation to nude mice [90]. Testicular cells derived from a leukemic rat can transmit lymphoblastic cells and subsequently induce leukemia even when as few as 20 cells are transplanted in a recipient rat [91]. Hitherto, successful removal of malignant cell types is difficult but of utmost importance for the success of SSCT. It is important to note that all these studies examine uncultured SSCs. Sorting procedures might be different in cultured cells as compared to uncultured cells, because expression of certain cell membrane markers is lost upon culturing. A careful selection of membrane markers still present on cultured SSCs or alternatively on tumor cells is important for efficient removal of malignant cells.

Besides the danger of reintroducing lingering malignant cells, the SSC culture system could also lead to the spontaneous arising of embryonic stem-like (ES-like) cells that are potentially carcinogenic. Indeed during the culture of mouse germ line cells, colonies of ES-like cells arise as spontaneous by-products of testicular cell cultures [92–94]. These ES-like cells are pluripotent and when they are relocated to an *in vivo* environment, they can form teratomas. Induction of teratomas after transplantation of testis derived ES-like cells has been well described in mouse models. In humans, the presence of pluripotent ES-like cells in germ line cultures is not as uniformly accepted as compared to mouse. Generation of ES-like colonies from human testis has been reported by several groups [95–97] but only one showed formation of teratomas that could be differentiated into cell types of all three germ layers [98]. Apart from this report, no other group could reproduce the formation of teratomas, and rather show that these testis-derived cell colonies do not express pluripotent markers at high levels. These two features are considered essential to classify cells as being pluripotent. Moreover, it has recently been shown that ES-like colonies in human germ line cultures have mesenchymal potential and might thus be multipotent rather than pluripotent [99, 100]. This also argues in favor of the theory that the “ES-like” colonies found in human germ line cultures are not truly ES-like. Teratoma formation upon accidental transplantation of ES-like colonies present in human germ line cultures is therefore less likely and seems of less significance in humans as compared to mice.

## 7. Health of Offspring 

Reports of SSCT-derived offspring mainly focus on the proof of concept that SSCT can generate offspring [29, 67–71, 101] while the general health of offspring is studied very minimally. Some studies perform no health analysis, while others only report basic variables such as weight, length [67] and fertility for a limited number of offspring [29, 68–70]. In some cases, growth abnormalities were observed in SSCT-derived offspring in mice [101]. It was shown that the karyotypes of first and second generation SSCT-conceived mice look normal as compared to naturally conceived mice [102]. The genome of F1 SSCT-conceived offspring was screened for genetic abnormalities by comparative genome hybridization (CGH) and no significant duplications or deletions were reported [70]. Remarkably, in some studies as many as 85–92% of all constructed embryos were lost during embryonic development [54, 101]. Whether this loss can be attributed to poor health of the embryos or technical constraint induced during round spermatid injection (ROSI) is unclear. ROSI has been associated with cleavage arrest in the early human embryo and similar failure rates have been described for generation of mouse embryos using ROSI [103, 104]. This suggests that the loss of embryos is caused by the ROSI procedure rather than SSC culturing or transplantation itself. CGH has the disadvantage that it can only reveal large genetic changes and cannot distinguish smaller genetic alterations such as SNPs or small CNVs. Base pair mutations on the single nucleotide level in spermatogonia have been shown to cause severe disease phenotypes including congenital disorders such as craniosynostosis syndrome (e.g., Apert Syndrome) [105, 106]. Since genetic alterations are essentially irreversible, they can be transmitted to the next generation and cause such phenotypes in the offspring. It is therefore crucial that the genome of experimental SSC derived offspring is screened on the highest resolution possible to reveal potentially harmful genetic mutations that arise in cultured SSCs.

In recent years, it has become apparent that epigenetic alterations may also be transmitted to subsequent generations. Studies on transgenerational epigenetic inheritance show the influence of the intrauterine environment on the epigenome and the mechanisms that lay behind these processes. Well-known examples of how the environment may cause heritable epimutations in humans are the Dutch Famine studies [107, 108] and the Överkalix cohort studies [109], which give evidence that both prenatal exposure to famine and food restriction during childhood are associated with an increased susceptibility of the offspring to multifactorial diseases such as cancer, diabetes, and cardiovascular disorders [56]. Animal studies have pointed out that various prenatal and early-life dietary conditions, such as high fat diet, low protein diet, overfeeding and malnutrition induce differential methylation of genes that may lead to a range of pathological phenotypes [110–116]. 

Methylation levels of the *H19* promoter and *Snrpn* promoters in SSCT-derived mice were shown to resemble those of naturally conceived controls, suggesting that there were no apparent methylation defects present in the offspring [70]. Likewise, no differences in DNA methylation of imprinted genes *Igf2* and *Peg1* occur in SSCT-conceived mice compared to naturally conceived mice [67]. In contradiction, distorted DNA methylation of *H19* and *Snrpn* promoter and altered histone modification was reported in pups conceived through fetal germ cell transplantation, alterations that were transmitted vertically up to 4 generations [101]. The observed epigenetic changes might be explained due to the potentially immature DNA methylation status of fetal germ cells compared to that of SSCs at the moment of isolation. During normal development, nearly all methylation marks undergo demethylation at the time PGCs migrate to the embryonic genital ridge and are remethylated in a sex-specific manner starting around the onset of the gonocyte stage [117]. Fetal germ cells may have been disrupted in the critical step of epigenetic reprogramming during culture, leading to the observed DNA methylation changes.

Heritable epigenetic influence on the phenotype is not only seen in experimental settings, but also in daily clinical care. Some studies show that assisted reproduction technologies (ART), such as IVF and ICSI, are associated with an increased risk of imprinting disorders such as Beckwith-Wiedemann Syndrome (BWS) [118]. Imprinting disorders are caused by loss or gain of parental DNA methylation at imprinted loci which results in aberrant gene expression during development and thereby leads to severe, irreversible phenotypic changes [119, 120]. The association between ART and imprinting disorders still remains controversial. While some studies on BWS show a 4-fold increased incidence of 4,6% in children conceived by IVF or ICSI in comparison to the background incidence rate of 0,8% [121], others report no increase of BWS cases in children conceived by ART [122, 123]. Moreover, it remains questionable whether the relation found between ART and imprinting disorders is causative, as it has been suggested that underlying sub-fertility of the parents might play a role [124]. Although SSCT and IVF/ICSI are both techniques to restore fertility, one should keep in mind that SSCT and IVF/ICSI are different on many levels. In SSCT resulting embryos are not cultured in an artificial environment, and therefore the risk to imprinting disorders may be absent or of a different magnitude than in IVF/ICSI. Even though the above results indicate that SSCT-derived offspring are fairly healthy, one must realize that the offspring studied are very low in number, in some cases as limited as one or two per study. 

## 8. Concluding Remarks

Ever since Brinster and Avarbock were able to obtain healthy offspring following SSCT in mice, many investigators have made efforts to translate this model into a clinical application. In this paper, we have discussed the current state of the art and hurdles that should be overcome (summarized in Figure 1) before SSCT can be implemented clinically. Many achievements have been made since the first successful transplantation in mice, and currently we are able to maintain and propagate human SSCs *in vitro *for long periods of time, without loss of expression of spermatogonial markers and with maintenance of their stem cell ability to migrate to the niche in the seminiferous tubules upon transplantation. These encouraging results make SSCT a potentially powerful therapeutic strategy to preserve and restore fertility in childhood cancer patients in the future. 

Future research needs to focus on a way to ensure there is no chance of reintroducing malignant cells in an individual that has just been treated and cured from a cancer. The risk of reintroducing malignancy by transplanting lingering tumor cells along with SSCs seems present as long as we cannot utterly remove them from cultures. Studies on the epigenetic stability of SSCs in culture and posttransplantation are scarce and results are contrasting. Efforts should be made to dissect the precise changes on both the genetic and epigenetic level when SSCs are cultured in an artificial environment. Adding up to this, it is unclear whether SSCT and subsequent SSC development to sperm from (epi-)genetically altered SSCs has any influence on the epigenome of the offspring. Arguably the most important factor is the health of offspring, and therefore more research should be started to assess general health of SSCT-conceived offspring in an adequate animal model with sufficiently large populations of animals, before a clinical trial in humans. 

Modern next-generation sequencing techniques make it more and more feasible to map the entire (epi-)genome of a cell culture or cell population on the single nucleotide level [125–127] and there are already a number of publications available that describe genome-wide DNA methylation for a range of male reproductive cell types [128, 129]. These advances provide us with a powerful tool to generate much needed information on how SSCs react to an *in vitro* culture environment in terms of methylation and base pair alterations. Steady progress concerning SSCT techniques is ongoing and this is why many researchers and clinicians are becoming increasingly confident that SSCT is viable as a way to restore fertility in prepubertal cancer patients. All in all, SSCT is a promising technique that will be beneficial for many young individuals diagnosed with cancer in the near future.

## Figures and Tables

**Table 1:** Selected milestones in the history of spermatogonial stem cell research.

Year	Author	Highlighted findings	Species	References
1966	Clermont	Initial histological description of A_pale_ and A_dark_ spermatogonia	Human	[35]
1971	Huckins	Model for renewal and differentiation of spermatogonia and existence of “spermatogonial stem cells” (SSCs)	Rat	[36]
1994	Brinster and Avarbock	First successful transplantation of testis-derived cells from one mouse to another resulting in donor-derived F1 progeny	Mouse	[18]
1998	Nagano et al.	*In vitro* maintenance of SSCs for 4 months on a somatic feeder layer	Mouse	[37]
1999	Schlatt et al.	Xenotransplantation of primate testis cell suspensions from one primate into the testes of another	Macaque	[23]
2002	Nagano et al.	First report on successful colonization of mouse testes after xenotransplanting human SSCs	Human	[38]
2003	Kanatsu-Shinohara et al.	Prolonged *in vitro* propagation of SSCs using GDNF, without immortalization of the cells in culture	Mouse	[29]
2005	Keros et al.	Proof of successful cryopreservation of testicular biopsies without decreasing structural integrity	Human	[39]
2005	Kanatsu-Shinohara et al.	Long-term propagation of SSCs under serum free and feeder free conditions	Mouse	[40]
2009	Sadri-Ardekani et al.	Long-term propagation of adult SSCs *in vitro* with retainment of functionality	Human	[31]
2011	Sadri-Ardekani et al.	Long-term propagation of prepubertal SSCs with retainment of functionality	Human	[30]
2012	Hermann et al.	Production of functional sperm by infertile prepubertal macaques after autotransplantation, capable of fertilizing oocytes	Macaque	[21]

## References

[1] Linabery AM, Ross JA (2008). Trends in childhood cancer incidence in the U.S. (1992–2004). *Cancer*.

[2] Kaatsch P, Pritchard-Jones K, Steliarova-Foucher E, Stiller CA, Coebergh JWW (2006). Cancer in children and adolescents in Europe: developments over 20 years and future challenges. *European Journal of Cancer*.

[3] Smith MA, Seibel NL, Altekruse SF (2010). Outcomes for children and adolescents with cancer: challenges for the twenty-first century. *Journal of Clinical Oncology*.

[4] Blatt J (1999). Pregnancy outcome in long-term survivors of childhood cancer. *Medical and Pediatric Oncology*.

[5] Oosterhuis BE, Goodwin T, Kiernan M, Hudson MM, Dahl GV (2008). Concerns about infertility risks among pediatric oncology patients and their parents. *Pediatric Blood and Cancer*.

[6] van Beek MEAB, Davids JAG, van de Kant HJG, de Rooij DG (1984). Response to fission neutron irradiation of spermatogonial stem cells in different stages of the cycle of the seminiferous epithelium. *Radiation Research*.

[7] van der Meer Y, Huiskamp R, Davids JAG, van der Tweel I, de Rooij DG (1992). The sensitivity of quiescent and proliferating mouse spermatogonial stem cells to X irradiation. *Radiation Research*.

[8] Singh SR, Burnicka-Turek O, Chauhan C, Hou SX (2011). Spermatogonial stem cells, infertility and testicular cancer. *Journal of Cellular and Molecular Medicine*.

[9] Brougham MFH, Kelnar CJH, Sharpe RM, Wallace WHB (2003). Male fertility following childhood cancer: current concepts and future therapies. *Asian Journal of Andrology*.

[10] Relander T, Cavallin-Ståhl E, Garwicz S, Olsson A, Willén M (2000). Gonadal and sexual function in men treated for childhood cancer. *Medical and Pediatric Oncology*.

[11] Rafsanjani KA, Faranoush M, Hedayatiasl AA, Vossough P (2007). Gonadal function and fertility in males survivors treated for Hodgkin’s disease in Iran. *Saudi Medical Journal*.

[12] Ginsberg JP, Carlson CA, Lin K (2010). An experimental protocol for fertility preservation in prepubertal boys recently diagnosed with cancer: a report of acceptability and safety. *Human Reproduction*.

[13] van den Berg H, Repping S, van der Veen F (2007). Parental desire and acceptability of spermatogonial stem cell cryopreservation in boys with cancer. *Human Reproduction*.

[14] Zebrack BJ, Casillas J, Nohr L, Adams H, Zeltzer LK (2004). Fertility issues for young adult survivors of childhood cancer. *Psycho-Oncology*.

[15] Langeveld NE, Grootenhuis MA, Voûte PA, de Haan RJ, van den Bos C (2004). Quality of life, self-esteem and worries in young adult survivors of childhood cancer. *Psycho-Oncology*.

[16] Devroey P, Liu J, Nagy Z (1995). Pregnancies after testicular sperm extraction and intracytoplasmic sperm injection in non-obstructive azoospermia. *Human Reproduction*.

[17] van Saen D, Tournaye H, Goossens E (2011). Presence of spermatogonia in 47, XXY men with no spermatozoa recovered after testicular sperm extraction. *Fertility and Sterility*.

[18] Brinster RL, Avarbock MR (1994). Germline transmission of donor haplotype following spermatogonial transplantation. *Proceedings of the National Academy of Sciences of the United States of America*.

[19] Brinster RL (2007). Male germline stem cells: from mice to men. *Science*.

[20] Kubota H, Brinster RL (2006). Technology insight: *in vitro* culture of spermatogonial stem cells and their potential therapeutic uses. *Nature Clinical Practice Endocrinology and Metabolism*.

[21] Hermann BP, Sukhwani M, Winkler F, Pascarella JN, Peters KA (2012). Spermatogonial stem cell transplantation into rhesus testes regenerates spermatogenesis producing functional sperm. *Cell Stem Cell*.

[22] Ning L, Meng J, Goossens E, Lahoutte T, Marichal M (2012). In search of an efficient injection technique for future clinical application of spermatogonial stem cell transplantation: infusion of contrast dyes in isolated cadaveric human testes. *Fertility and Sterility*.

[23] Schlatt S, Rosiepen G, Weinbauer GF, Rolf C, Brook PF, Nieschlag E (1999). Germ cell transfer into rat, bovine, monkey and human testes. *Human Reproduction*.

[24] Geens M, de Block G, Goossens E, Frederickx V, van Steirteghem A, Tournaye H (2006). Spermatogonial survival after grafting human testicular tissue to immunodeficient mice. *Human Reproduction*.

[25] Mitchell RT, Saunders PTK, Childs AJ (2010). Xenografting of human fetal testis tissue: a new approach to study fetal testis development and germ cell differentiation. *Human Reproduction*.

[26] Sato T, Katagiri K, Yokonishi T, Kubota Y, Inoue K (2011). *In vitro* production of fertile sperm from murine spermatogonial stem cell lines. *Nature Communications*.

[27] Yang S, Bo J, Hu H, Guo X, Tian R (2012). Derivation of male germ cells from induced pluripotent stem cells *in vitro* and in reconstituted seminiferous tubules. *Cell Proliferation*.

[28] Tegelenbosch RAJ, de Rooij DG (1993). A quantitative study of spermatogonial multiplication and stem cell renewal in the C3H/101 F1 hybrid mouse. *Mutation Research*.

[29] Kanatsu-Shinohara M, Ogonuki N, Inoue K (2003). Long-term proliferation in culture and germline transmission of mouse male germline stem cells. *Biology of Reproduction*.

[30] Sadri-Ardekani H, Akhondi MA, van der Veen F, Repping S, van Pelt AMM (2011). *In vitro* propagation of human prepubertal spermatogonial stem cells. *The Journal of the American Medical Association*.

[31] Sadri-Ardekani H, Mizrak SC, van Daalen SKM (2009). Propagation of human spermatogonial stem cells *in vitro*. *The Journal of the American Medical Association*.

[32] Kubota H, Avarbock MR, Brinster RL (2004). Growth factors essential for self-renewal and expansion of mouse spermatogonial stem cells. *Proceedings of the National Academy of Sciences of the United States of America*.

[33] Nagano M, Ryu BY, Brinster CJ, Avarbock MR, Brinster RL (2003). Maintenance of mouse male germ line stem cells *in vitro*. *Biology of Reproduction*.

[34] Ebata KT, Yeh JR, Zhang X, Nagano MC (2011). Soluble growth factors stimulate spermatogonial stem cell divisions that maintain a stem cell pool and produce progenitors *in vitro*. *Experimental Cell Research*.

[35] Clermont Y (1966). Renewal of spermatogonia in man. *American Journal of Anatomy*.

[36] Huckins C (1971). The spermatogonial stem cell population in adult rats. I. Their morphology, proliferation and maturation. *Anatomical Record*.

[37] Nagano M, Avarbock MR, Leonida EB, Brinster CJ, Brinster RL (1998). Culture of mouse spermatogonial stem cells. *Tissue and Cell*.

[38] Nagano M, Patrizio P, Brinster RL (2002). Long-term survival of human spermatogonial stem cells in mouse testes. *Fertility and Sterility*.

[39] Keros V, Rosenlund B, Hultenby K, Aghajanova L, Levkov L, Hovatta O (2005). Optimizing cryopreservation of human testicular tissue: comparison of protocols with glycerol, propanediol and dimethylsulphoxide as cryoprotectants. *Human Reproduction*.

[40] Kanatsu-Shinohara M, Miki H, Inoue K (2005). Long-term culture of mouse male germline stem cells under serum- or feeder-free conditions. *Biology of Reproduction*.

[41] Creemers LB, den Ouden K, van Pelt AMM, de Rooij DG (2002). Maintenance of adult mouse type A spermatogonia *in vitro*: influence of serum and growth factors and comparison with prepubertal spermatogonial cell culture. *Reproduction*.

[42] Mirzapour T, Movahedin M, Tengku Ibrahim TA, Koruji M, Haron AW (2011). Effects of basic fibroblast growth factor and leukaemia inhibitory factor on proliferation and short-term culture of human spermatogonial stem cells. *Andrologia*.

[43] Kanatsu-Shinohara M, Takashima S, Ishii K, Shinohara T (2011). Dynamic changes in EPCAM expression during spermatogonial stem cell differentiation in the mouse testis. *PLOS ONE*.

[44] McCarroll SA, Altshuler DM (2007). Copy-number variation and association studies of human disease. *Nature Genetics*.

[45] Mefford HC, Sharp AJ, Baker C (2008). Recurrent rearrangements of chromosome 1q21.1 and variable pediatric phenotypes. *The New England Journal of Medicine*.

[46] Vissers LELM, Veltman JA, van Kessel AG, Brunner HG (2005). Identification of disease genes by whole genome CGH arrays. *Human Molecular Genetics*.

[47] Foudah D, Redaelli S, Donzelli E (2009). Monitoring the genomic stability of *in vitro* cultured rat bone-marrow-derived mesenchymal stem cells. *Chromosome Research*.

[48] Maitra A, Arking DE, Shivapurkar N (2005). Genomic alterations in cultured human embryonic stem cells. *Nature Genetics*.

[49] Liu AM, Qu WW, Liu X, Qu CK (2012). Chromosomal instability in *in vitro* cultured mouse hematopoietic cells associated with oxidative stress. *American Journal of Blood Research*.

[50] Thonneau P, Bujan L, Multigner L, Mieusset R (1998). Occupational heat exposure and male fertility: a review. *Human Reproduction*.

[51] Banks S, King SA, Irvine DS, Saunders PTK (2005). Impact of a mild scrotal heat stress on DNA integrity in murine spermatozoa. *Reproduction*.

[52] Baker DEC, Harrison NJ, Maltby E (2007). Adaptation to culture of human embryonic stem cells and oncogenesis *in vivo*. *Nature Biotechnology*.

[53] Zhou YF, Bosch-Marce M, Okuyama H (2006). Spontaneous transformation of cultured mouse bone marrow-derived stromal cells. *Cancer Research*.

[54] Kanatsu-Shinohara M, Ogonuki N, Iwano T (2005). Genetic and epigenetic properties of mouse male germline stem cells during long-term culture. *Development*.

[55] Baird DM, Britt-Compton B, Rowson J, Amso NN, Gregory L, Kipling D (2006). Telomere instability in the male germline. *Human Molecular Genetics*.

[56] Skinner MK (2011). Environmental epigenomics and disease susceptibility. *EMBO Reports*.

[57] Weinhouse C, Anderson O, Jones T, Kim J, Liberman S (2011). An expression microarray approach for the identification of metastable epialleles in the mouse genome. *Epigenetics*.

[58] Berger SL, Kouzarides T, Shiekhattar R, Shilatifard A (2009). An operational definition of epigenetics. *Genes and Development*.

[59] Cedar H, Bergman Y (2009). Linking DNA methylation and histone modification: patterns and paradigms. *Nature Reviews Genetics*.

[60] Feinberg AP, Vogelstein B (1983). Hypomethylation distinguishes genes of some human cancers from their normal counterparts. *Nature*.

[61] Hansen KD, Timp W, Bravo HC (2011). Increased methylation variation in epigenetic domains across cancer types. *Nature Genetics*.

[62] Bork S, Pfister S, Witt H (2010). DNA methylation pattern changes upon long-term culture and aging of human mesenchymal stromal cells. *Aging Cell*.

[63] Schellenberg A, Lin Q, Schuler H, Koch CM, Joussen S (2011). Replicative senescence of mesenchymal stem cells causes DNA-methylation changes which correlate with repressive histone marks. *Aging*.

[64] Goossens E, Bilgec T, van Saen D, Tournaye H (2011). Mouse germ cells go through typical epigenetic modifications after intratesticular tissue grafting. *Human Reproduction*.

[65] Nix DA, Hammoud SS, Zhang H, Purwar J, Carrell DT, Cairns BR (2009). Distinctive chromatin in human sperm packages genes for embryo development. *Nature*.

[66] Oh SH, Jung YH, Gupta MK, Uhm SJ, Lee HT (2009). H19 gene is epigenetically stable in mouse multipotent germline stem cells. *Molecules and Cells*.

[67] Goossens E, de Rycke M, Haentjens P, Tournaye H (2009). DNA methylation patterns of spermatozoa and two generations of offspring obtained after murine spermatogonial stem cell transplantation. *Human Reproduction*.

[68] Kubota H, Avarbock MR, Schmidt JA, Brinster RL (2009). Spermatogonial stem cells derived from infertile W^v^/W^v^ mice self-renew *in vitro* and generate progeny following transplantation. *Biology of Reproduction*.

[69] Ryu BY, Orwig KE, Oatley JM (2007). Efficient generation of transgenic rats through the male germline using lentiviral transduction and transplantation of spermatogonial stem cells. *Journal of Andrology*.

[70] Wu X, Goodyear SM, Abramowitz LK, Bartolomei MS, Tobias JW (2012). Fertile offspring derived from mouse spermatogonial stem cells cryopreserved for more than 14 years. *Human Reproduction*.

[71] Yuan Z, Hou R, Wu J (2009). Generation of mice by transplantation of an adult spermatogonial cell line after cryopreservation. *Cell Proliferation*.

[72] Honaramooz A, Megee SO, Dobrinski I (2002). Germ cell transplantation in pigs. *Biology of Reproduction*.

[73] den Ouden K, Izadyar F, Creemers LB, Posthuma G, Parvinen M, de Rooij DG (2003). Proliferation and differentiation of bovine type A spermatogonia during long-term culture. *Biology of Reproduction*.

[74] Kawasaki T, Saito K, Sakai C, Shinya M, Sakai N (2012). Production of zebrafish offspring from cultured spermatogonial stem cells. *Genes to Cells*.

[75] Nóbrega RH, Greebe CD, van de Kant H, Bogerd J, de França LR, Schulz RW (2010). Spermatogonial stem cell niche and spermatogonial stem cell transplantation in zebrafish. *PLOS ONE*.

[76] Dobrinski I, Avarbock MR, Brinster RL (1999). Transplantation of germ cells from rabbits and dogs into mouse testes. *Biology of Reproduction*.

[77] Ogawa T, Dobrinski I, Avarbock MR, Brinster RL (1999). Xenogeneic spermatogenesis following transplantation of hamster germ cells to mouse testes. *Biology of Reproduction*.

[78] Aponte PM, Soda T, Teerds KJ, Mizrak SC, van de Kant HJG, de Rooij DG (2008). Propagation of bovine spermatogonial stem cells *in vitro*. *Reproduction*.

[79] Izadyar F, den Ouden K, Creemers LB, Posthuma G, Parvinen M, de Rooij DG (2003). Proliferation and differentiation of bovine type A spermatogonia during long-term culture. *Biology of Reproduction*.

[80] Oatley JM, de Avila DM, McLean DJ, Griswold MD, Reeves JJ (2002). Transplantation of bovine germinal cells into mouse testes. *Journal of Animal Science*.

[81] Rajpert-De Meyts E, Hanstein R, Jørgensen N, Graem N, Vogt PH, Skakkebæk NE (2004). Developmental expression of *POU5F1* (OCT-3/4) in normal and dysgenetic human gonads. *Human Reproduction*.

[82] Mitchell RT, Cowan G, Morris KD (2008). Germ cell differentiation in the marmoset (*Callithrix jacchus*) during fetal and neonatal life closely parallels that in the human. *Human Reproduction*.

[83] Jahnukainen K, Ehmcke J, Nurmio M, Schlatt S (2012). Autologous ectopic grafting of cryopreserved testicular tissue preserves the fertility of prepubescent monkeys that receive sterilizing cytotoxic therapy. *Cancer Research*.

[84] Nagano M, McCarrey JR, Brinster RL (2001). Primate spermatogonial stem cells colonize mouse testes. *Biology of Reproduction*.

[85] Radford JA (2000). Is prevention of sterility possible in men?. *Annals of Oncology*.

[86] Akhtar M, Ali MA, Burgess A, Aur RJ (1991). Fine-needle aspiration biopsy (FNAB) diagnosis of testicular involvement in acute lymphoblastic leukemia in children. *Diagnostic Cytopathology*.

[87] García-González R, Pinto J, Val-Bernal JF (2000). Testicular metastases from solid tumors: an autopsy study. *Annals of Diagnostic Pathology*.

[88] Tsujimura A, Fujita K, Miyagawa Y (2006). Isolation of germ cells from leukemia and lymphoma cells in a human *in vitro* model: potential clinical application for restoring human fertility after anticancer therapy. *Cancer Research*.

[89] Geens M, van de Velde H, de Block G, Goossens E, van Steirteghem A, Tournaye H (2007). The efficiency of magnetic-activated cell sorting and fluorescence-activated cell sorting in the decontamination of testicular cell suspensions in cancer patients. *Human Reproduction*.

[90] Hermann BP, Sukhwani M, Salati J, Sheng Y, Chu T (2011). Separating spermatogonia from cancer cells in contaminated prepubertal primate testis cell suspensions. *Human Reproduction*.

[91] Jahnukainen K, Hou M, Petersen C, Setchell B, Söder O (2001). Intratesticular transplantation of testicular cells from leukemic rats causes transmission of leukemia. *Cancer Research*.

[92] Guan K, Nayernia K, Maier LS (2006). Pluripotency of spermatogonial stem cells from adult mouse testis. *Nature*.

[93] Inoue K, Kanatsu-Shinohara M, Lee J (2004). Generation of pluripotent stem cells from neonatal mouse testis. *Cell*.

[94] Seandel M, James D, Shmelkov SV (2007). Generation of functional multipotent adult stem cells from GPR125^+^ germline progenitors. *Nature*.

[95] Golestaneh N, Kokkinaki M, Pant D (2009). Pluripotent stem cells derived from adult human testes. *Stem Cells and Development*.

[96] Kossack N, Meneses J, Shefi S (2009). Isolation and characterization of pluripotent human spermatogonial stem cell-derived cells. *Stem Cells*.

[97] Mizrak SC, Chikhovskaya JV, Sadri-Ardekani H (2010). Embryonic stem cell-like cells derived from adult human testis. *Human Reproduction*.

[98] Conrad S, Renninger M, Hennenlotter J (2008). Generation of pluripotent stem cells from adult human testis. *Nature*.

[99] Chikhovskaya J, Jonker M, Meissner A, Breit T, Repping S (2012). Human testis-derived embryonic stem cell-like cells are not pluripotent, but possess potential of mesenchymal progenitors. *Human Reproduction*.

[100] Ko K, Reinhardt P, Tapia N (2011). Brief report: evaluating the potential of putative pluripotent cells derived from human testis. *Stem Cells*.

[101] Lee J, Kanatsu-Shinohara M, Ogonuki N (2009). Heritable imprinting defect caused by epigenetic abnormalities in mouse spermatogonial stem cells. *Biology of Reproduction*.

[102] Goossens E, de Vos P, Tournaye H (2010). Array comparative genomic hybridization analysis does not show genetic alterations in spermatozoa and offspring generated after spermatogonial stem cell transplantation in the mouse. *Human Reproduction*.

[103] Levran D, Nahum H, Farhi J, Weissman A (2000). Poor outcome with round spermatid injection in azoospermic patients with maturation arrest. *Fertility and Sterility*.

[104] Ogonuki N, Inoue K, Ogura A (2011). Birth of normal mice following round spermatid injection without artificial oocyte activation. *Journal of Reproduction and Development*.

[105] Goriely A, McVean GAT, van Pelt AMM (2005). Gain-of-function amino acid substitutions drive positive selection of *FGFR2* mutations in human spermatogonia. *Proceedings of the National Academy of Sciences of the United States of America*.

[106] Yu K, Herr AB, Waksman G, Ornitz DM (2000). Loss of fibroblast growth factor receptor 2 ligand-binding specificity in Apert syndrome. *Proceedings of the National Academy of Sciences of the United States of America*.

[107] Botden IP, Zillikens MC, de Rooij SR, Langendonk JG, Danser AH (2012). Variants in the SIRT1 gene may affect diabetes risk in interaction with prenatal exposure to famine. *Diabetes Care*.

[108] de Rooij SR, Painter RC, Phillips DIW (2006). Impaired insulin secretion after prenatal exposure to the Dutch famine. *Diabetes Care*.

[109] Pembrey ME, Bygren LO, Kaati G (2006). Sex-specific, male-line transgenerational responses in humans. *European Journal of Human Genetics*.

[110] Bogdarina I, Haase A, Langley-Evans S, Clark AJL (2010). Glucocorticoid effects on the programming of AT1b angiotensin receptor gene methylation and expression in the rat. *PLoS ONE*.

[111] Jiménez-Chillarón JC, Díaz R, Martínez D, Pentinat T, Ramón-Krauel M (2012). The role of nutrition on epigenetic modifications and their implications on health. *Biochimie*.

[112] MacLennan NK, James SJ, Melnyk S (2004). Uteroplacental insufficiency alters DNA methylation, one-carbon metabolism, and histone acetylation in IUGR rats. *Physiological Genomics*.

[113] Plagemann A, Harder T, Brunn M (2009). Hypothalamic proopiomelanocortin promoter methylation becomes altered by early overfeeding: an epigenetic model of obesity and the metabolic syndrome. *The Journal of Physiology*.

[114] Thompson RF, Fazzari MJ, Niu H, Barzilai N, Simmons RA, Greally JM (2010). Experimental intrauterine growth restriction induces alterations in DNA methylation and gene expression in pancreatic islets of rats. *Journal of Biological Chemistry*.

[115] van Straten EME, Bloks VW, Huijkman NCA (2010). The liver X-receptor gene promoter is hypermethylated in a mouse model of prenatal protein restriction. *American Journal of Physiology*.

[116] Vucetic Z, Kimmel J, Reyes TM (2011). Chronic high-fat diet drives postnatal epigenetic regulation of *μ*-opioid receptor in the brain. *Neuropsychopharmacology*.

[117] Daxinger L, Whitelaw E (2012). Understanding transgenerational epigenetic inheritance via the gametes in mammals. *Nature Reviews Genetics*.

[118] Zechner U, Pliushch G, Schneider E (2009). Quantitative methylation analysis of developmentally important genes in human pregnancy losses after ART and spontaneous conception. *Molecular Human Reproduction*.

[119] Kobayashi H, Hiura H, John RM (2009). DNA methylation errors at imprinted loci after assisted conception originate in the parental sperm. *European Journal of Human Genetics*.

[120] Kobayashi H, Sato A, Otsu E (2007). Aberrant DNA methylation of imprinted loci in sperm from oligospermic patients. *Human Molecular Genetics*.

[121] DeBaun MR, Niemitz EL, Feinberg AP (2003). Association of *in vitro* fertilization with Beckwith-Wiedemann syndrome and epigenetic alterations of LIT1 and H19. *American Journal of Human Genetics*.

[122] Källén B, Finnström O, Nygren KG, Olausson PO (2005). *In vitro* fertilization (IVF) in Sweden: infant outcome after different IVF fertilization methods. *Fertility and Sterility*.

[123] Lidegaard Ø, Pinborg A, Andersen AN (2005). Imprinting diseases and IVF: Danish National IVF cohort study. *Human Reproduction*.

[124] Filipponi D, Feil R (2009). Perturbation of genomic imprinting in oligozoospermia. *Epigenetics*.

[125] Bernstein BE, Birney E, Dunham I, Green ED, Gunter C (2012). An integrated encyclopedia of DNA elements in the human genome. *Nature*.

[126] Mardis ER (2008). Next-generation DNA sequencing methods. *Annual Review of Genomics and Human Genetics*.

[127] Metzker ML (2010). Sequencing technologies—the next generation. *Nature Reviews Genetics*.

[128] Meissner A, Mikkelsen TS, Gu H (2008). Genome-scale DNA methylation maps of pluripotent and differentiated cells. *Nature*.

[129] Smith ZD, Chan MM, Mikkelsen TS, Gu H, Gnirke A, " (2012). A unique regulatory phase of DNA methylation in the early mammalian embryo. *Nature*.

